# Thermal Model and Countermeasures for Future Smart Glasses †

**DOI:** 10.3390/s20051446

**Published:** 2020-03-06

**Authors:** Kodai Matsuhashi, Toshiki Kanamoto, Atsushi Kurokawa

**Affiliations:** Graduate School of Science and Technology, Hirosaki University, Aomori 036-8560, Japan; ms19518@eit.hirosaki-u.ac.jp (K.M.); kana@hirosaki-u.ac.jp (T.K.)

**Keywords:** thermal management, wearable device, thermal modeling, smart glasses, thermal analysis

## Abstract

The market for wearable devices such as smart watches and smart glasses continues to grow rapidly. Smart glasses are attracting particular attention because they offer convenient features such as hands-free augmented reality (AR). Since smart glasses directly touch the face and head, the device with high temperature has a detrimental effect on human physical health. This paper presents a thermal network model in a steady state condition and thermal countermeasure methods for thermal management of future smart glasses. It is accomplished by disassembling the state by wearing smart glasses into some parts, creating the equivalent thermal resistance circuit for each part, approximating heat-generating components such as integrated circuits (ICs) to simple physical structures, setting power consumption to the heat sources, and providing heat transfer coefficients of natural convection in air. The average temperature difference between the thermal network model and a commercial thermal solver is 0.9 °C when the maximum temperature is 62 °C. Results of an experiment using the model show that the temperature of the part near the ear that directly touches the skin can be reduced by 51.4% by distributing heat sources into both sides, 11.1% by placing higher heat-generating components farther from the ear, and 65.3% in comparison with all high conductivity materials by using a combination of low thermal conductivity materials for temples and temple tips and high conductivity materials for rims.

## 1. Introduction

Wearable devices have become popular as state-of-the-art electronic devices, such as smart watches, smart glasses, smart clothing, and fitness trackers, have been made commercially available for consumer and industrial uses. Currently, smart watches are the wearable device with the largest market size. However, smart glasses have also been released by many companies [1,2,3,4,5,6,7] and can be used for various purposes such as medical care, health, learning/education, and entertainment. Differences in the uses between the smart glasses and the wrist-worn wearables such as smart watches and fitness trackers come from the differences between wrists and eyes. Smart glasses have advantages that users can look at various things such as maps (e.g., current location) and movies with augmented reality (AR) through a display, and their eye and facial movements can be recognized for medical care, health monitoring, and dozing prevention. In the future, heat issues will become more serious because smart glasses will require faster central processing unit (CPU) and larger memory to deal with enormous amounts of data. Therefore, thermal design is becoming one of the key technologies for future wearable devices.

Various techniques for utilizing smart glasses have been developed by many researchers. For medical uses, techniques have been presented for clinical and surgical applications [8], medical emergency situations [9], and disaster medicine [10]. For recognition and interface, techniques have been presented for head gestures [11], face detection [12], eye movements [13], user authentication [14], estimation of respiration rate [15], speech interaction with eye blinking detection [16], context-aware lightning control [17], distance learning [18], and indoor localization [19], drowsiness and fatigue detection to increase road safety [20], countermeasures to phishing attacks [21], gait aid for Parkinson’s disease patients [22], contextually-aware learning in physics experiments [23], and guiding for visually impaired users [24]. Some techniques regarding thermals have been presented such as 3D thermal model reconstruction based on image-based modeling using smartphone sensors [25], design of an oven utilizing radiative heat transfer for smart phone panels [26], thermal management systems for civil aircraft engines [27], and thermal properties of glasses [28]. A system for low-power smart glasses has been presented [29]. However, there have been only a few technical reports about the heat of smart glasses [30,31]. Smart glasses directly touch human skin. The heat of the smart glasses is capable of causing burns of the skin. Thermal management of smart glasses is essential for physical health safety and comfortable use. Current smart glasses consume 1 to 3 W under various workloads [30]. We presented thermal countermeasures of smart glasses [31] and discussed only the maximum temperature in the integrated circuits that generate heat. Only a heat generating component was used and was placed on a limited space. Moreover, the temperature of the device surface touching skin that may cause a low-temperature burn even at 43 °C [32] was not analyzed. In Reference [33], we presented a thermal network model for thermal designs of future smart glasses. In this paper, we provide more detailed resistance models for all parts of devices, thermal properties that were used in the analysis, a difference in temperatures due to the position on the temple, more detailed explanations for each figure, motivations for this work, and discussions.

Smart glasses mainly comprise the electronic device body, and display a liquid crystal on the silicon (LCOS) device [34] and battery. The device body consists of many heat generating electronic components, including processors, memory, wireless modules, and power management integrated circuits (ICs). In accordance with a design concept, the device body is mounted to various places [35]. We simplify the device body as follows: IC packages are reconstructed by using three layers (heat generating, upper, and lower) in the vertical direction, and several packages are arranged horizontally on a printed circuit board (PCB). Thereby, the simplified device body can be re-sized and consume power in a non-uniform manner. By making a thermal resistance model of the state wearing smart glasses, temperatures at each part can be calculated. The thermal model can be used for various types of smart glasses such as glasses’ structures, materials, heat sources, and layouts of the components.

Smart glasses include various functions such as a camera, video, map, translation, weather information, and search in real-world environments, augmented reality, and virtual reality (VR). As the demand for higher precision and higher speed increases, power consumption also increases. Moreover, power density increases with higher integration (including 3D ICs). Therefore, thermal management is very important for future smart glasses. Systems and design methodologies of smart glasses have been proposed [29,36,37,38,39]. Among them, thermal management has become one of the crucial issues in AR and VR processing [30,31] where image screens as well as image sensors have been equipped. Even the current high definition (HD) smart glasses consume 1 to 3 W [30]. Upcoming advanced features including 4K/8K resolution processing are expected to need additional power to render the images [40,41,42,43,44,45]. This will require more organized thermal management with overviewing packages, boards, and systems as well as heating processor chips [46]. Motion detection is another power consuming factor. Even the current artificial intelligence (AI)-based moving object identification from the sensor images also requires up to 3W of power. Additionally, the expected features in the near future such as human detection will impose an extra power expense. Furthermore, promising smart glasses need to communicate with external networks and transfer large amounts of the processed data. The leading 5G communications technologies reduce transmission power in exchange for consuming additional circuit power due to the required hardware expansions including signal processing to establish low-power mmWave communications at extremely high frequencies [47].

In the viewpoint of the safety and comfort of smart glasses, there are several issues such as weight [48,49], battery [50,51], AR/VR [52,53,54], and heat [31,33]. In Reference [48], regarding weight issues, the effect of weight balance for shutter glasses in terms of subjective discomfort and physical load on the nose has been investigated [48] and a user discomfort on the different wearing mode glasses with different support points has been presented [49]. With respect to battery safety, an extremely safe and wearable solid-state zinc ion battery has been fabricated [50] and a quasi-solid-state aqueous rechargeable lithium-ion battery with outstanding stability, flexibility, safety, and breathability toward various wearable electronics has been reported [51]. AR/VR include many problems such as display size, resolution, computing capability, negative effects in some people with autism spectrum disorder, and architecture/server/network issues [52,53,54]. Moreover, it is necessary to ensure the safety and comfort due to heat generation [31,33] since smart glasses are worn on the face/head of the human body for use.

The main sources of heat generation of smart glasses come from power consumptions of ICs. Thermal countermeasures within IC chips must be mainly low power designs and have a limit. For smart glasses, the temperature not only in chips for circuit operation but also on the device surface touching the skin is important for preventing low temperature burns. Thermal management of smart glasses is required to determine various conditions such as arrangements of heat generating components and materials of parts. Although a commercial thermal solver can obtain high accuracy results, it has the disadvantages of a complex structure input, long processing time, and unsuitability for parameter optimization. Therefore, we have developed a thermal network model to improve design efficiency. The model has been devised for not only current products but also future products. To deal with as wide a variety of smart glasses as possible, the entire thermal network is divided into several parts and is expressed by a block diagram (as described in Section 3.1). In addition, each block is removable and replaceable. We use virtual smart glasses composed of some parts but not real smart glasses so that the entire thermal network can be applied to various types of smart glasses.

Additionally, using the proposed model, we present thermal countermeasures of smart glasses for ensuring the health safety and comfortable use. We clarify the following facts: (1) If high thermal conductivity materials like Al are used for a grasses frame, the whole temperature can be reduced, but a low temperature burn may be caused near an ear. (2) If low conductivity materials like cellulose acetate (CA) plastic are used, temperature near an ear can be reduced, but the surface temperature of the device body rises. (3) When Al is used, by locating higher power density, ICs near the lens, temperature at the ear decreases but not sufficiently. (4) When the device body is divided and placed on both sides, temperatures decrease as a whole. From these results, we found that the best solution is to use plastic for the temples and temple tips for hanging on the ears and Al for the other parts of the frame in order to locate the device body to the lens side and divide it into both sides as much as possible.

The rest of the paper is organized as follows. Section 2 describes the details of smart glasses assumed in this work. Section 3 presents thermal network models for the smart glasses. Section 4 shows experimental results for thermal countermeasures. Section 5 presents discussions of this work. Section 6 concludes this paper.

## 2. Physical Structural Model of Smart Glasses

In this section, the smart glasses assumed in this work are described. First, an overview of the smart glasses is shown. Next, a physical structural model with dimensions is presented. Lastly, the heat generating components are discussed.

### 2.1. Overview of Smart Glasses

**Figure 1:**
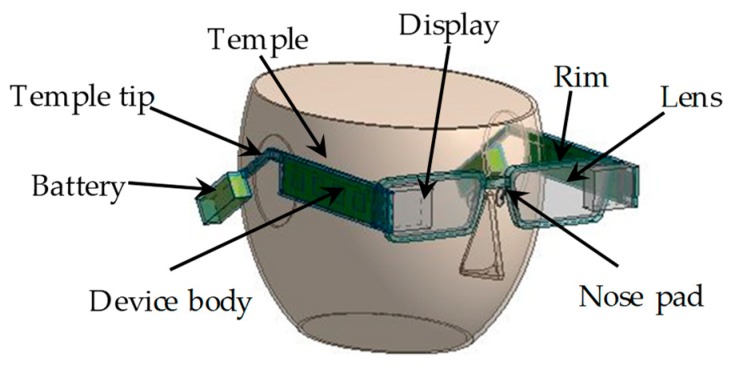
Overview of smart glasses used in this paper.

Figure 1 shows an overview of a basic structure of the smart glasses used in this study. In the basic structure of smart glasses, batteries are connected to device bodies. Device body cases are mounted on the temples of both sides. Electronic components are installed in the right device body, and displays are set in front of lenses.

### 2.2. Structure of Smart Glasses

**Figure 2:**
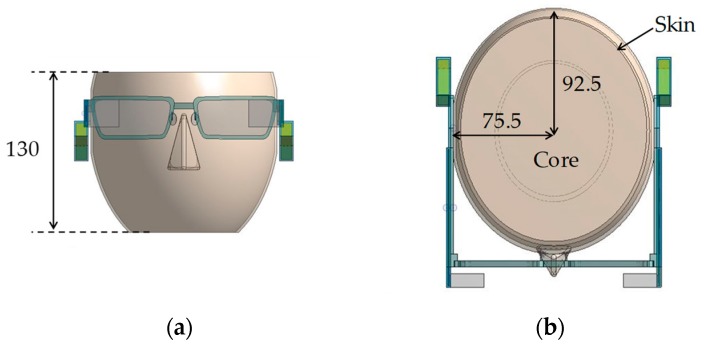
Overview of smart glasses used in this paper: (**a**) front view and (**b**) top view.

**Figure 3:**
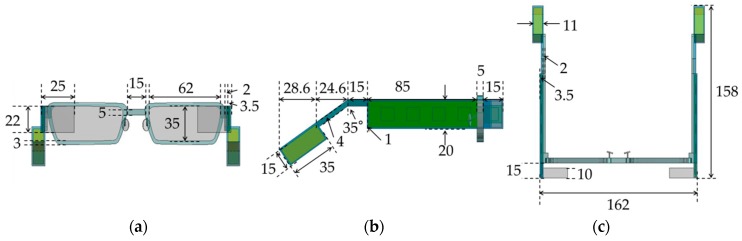
Dimensions (in mm) of smart glasses: (**a**) front view, (**b**) side view, and (**c**) top view.

Table 1 lists thermal properties of the smart glasses used in our basic analysis. Figure 2 shows dimensions of a face/head model. For a face model, we referred to a model of human thermoregulation [55] and used a simpler model. The skin thickness was 2 mm. The core and ambient temperatures were set to 36.6 and 25 °C, respectively. Figure 3 shows dimensions of the smart glasses.

### 2.3. Heat Generating Components

**Figure 4:**
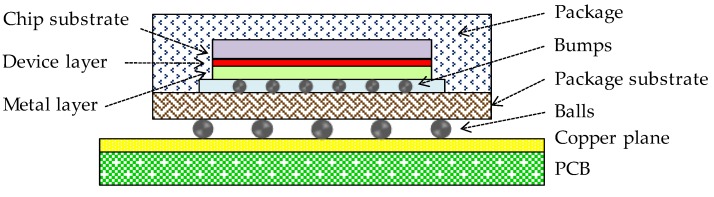
IC package (flip-chip package).

**Figure 5:**
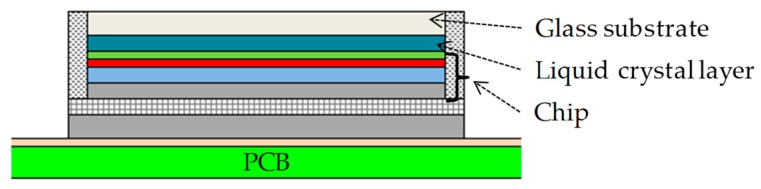
Liquid crystal on the silicon (LCOS) device.

In general, heat generating components of smart glasses include processors, memories (e.g., DDR4 SDRAM, and NAND flash), audio ICs, wireless modules, power management ICs, and LCOS devices. Figure 4 illustrates an example of a cross-sectional structure of an IC package with the flip-chip technology. Table 2 lists an example of the thermal property and thickness of each layer. Figure 5 illustrates an example of a cross-sectional structure of an LCOS device. Table 3 lists an example of the thermal property and size. The heat generating components are composed of various structures and thermal properties.

**Figure 6:**
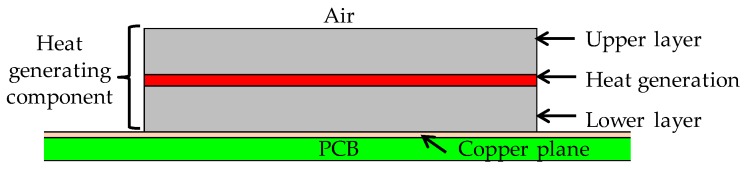
Simple model of the heat generating component.

In this scenario, we model the heat generating components by applying them to various types of smart glasses. The simple physical model for an equivalent circuit of one heat generating component is approximated with upper and lower layers, as shown in Figure 6. 

**Figure 7:**
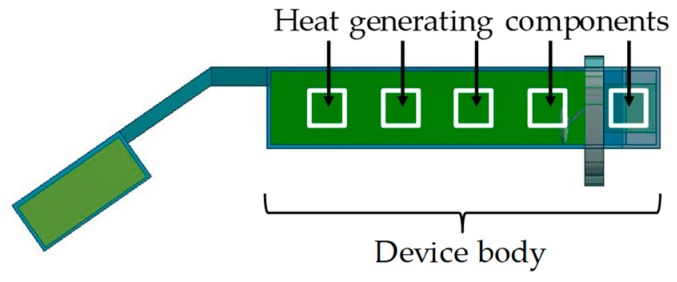
Layout of heat generating components.

In this work, in the device body at one side, we assumed the use of four IC packages shown in Figure 4 with conditions in Table 2 and one LCOS device shown in Figure 5 with conditions in Table 3. Figure 7 shows the arrangement of five heat generating components. A thermal resistance network for the device body with their components is constructed by using the simple physical model in Figure 6.

### 2.4. Structures and Materials of Components

In the future, smart glasses with various shapes, size, materials, and arrangements of components must be produced. Size and materials are basically modifiable because they can be applied by changing thermal resistance values.

**Figure 8:**
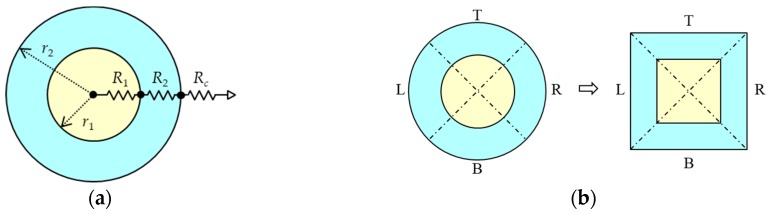
Thermal modeling in cross sections for cylindrical battery: (**a**) thermal resistance circuit and (**b**) conversion of the circle to rectangle.

Although this paper uses rectangular lithium polymer (LiPo) batteries, the shapes and materials of batteries are not greatly restricted because a battery is modeled simply. In this case, thermal resistance circuits for a cylindrical battery are discussed. Figure 8a shows a cross section of a cylindrical battery. A cylinder is expressed with three thermal resistances [56,57]. The inner liquid fluid in a battery is covered by a frame. The heat conduction resistance value for the internal cylinder (inner liquid fluid) can be calculated from the equation below.

(1) $$R_{1}=\frac{ln\left(r_{1}\right)}{2\pi kl}$$

where *r*_1_ is the internal radius, *k* is the thermal conductivity of the material, and *l* is the length of a cylinder. The heat conduction resistance value for an outer frame can be calculated from the equation below.

(2) $$R_{2}=\frac{ln\left(r_{2}/r_{1}\right)}{2\pi kl}$$

where *r*_2_ is the outer radius. The heat convection resistance value from a frame surface can be calculated from Equation (3) below.

(3) $$R_{c}=\frac{1}{h_{c}2\pi r_{2}l}$$

where *h*_c_ is the heat transfer coefficient. In this way, all the necessary resistances can be obtained. By assigning them the model of rectangular batteries (i.e., by converting a circle into a rectangle as shown in Figure 8b), a cylindrical battery is also applicable.

For modeling heat generating components, we used the PCB with a flip chip-ball grid array (FC-BGA) package shown in Figure 4 as an example. However, types of packages (e.g., wafer level package (WLP)) and boards are not greatly restricted because a heat generating component is modelled very simply as a structure shown in Figure 6. The idea of the thermal modeling presented in this paper can use not only rigid boards but also flexible substrates [58,59,60] such as polyimide and polyethylene terephthalate (PET). However, if such flexible circuits are used, the model of device body parts should be replaced with a more appropriate thermal model.

## 3. Thermal Network Model

In this section, we present a thermal network model for smart glasses in a steady state condition. Based on a block diagram for an entire thermal network, thermal models for each block are presented.

### 3.1. Block Diagram for Entire Thermal Network

**Figure 9:**
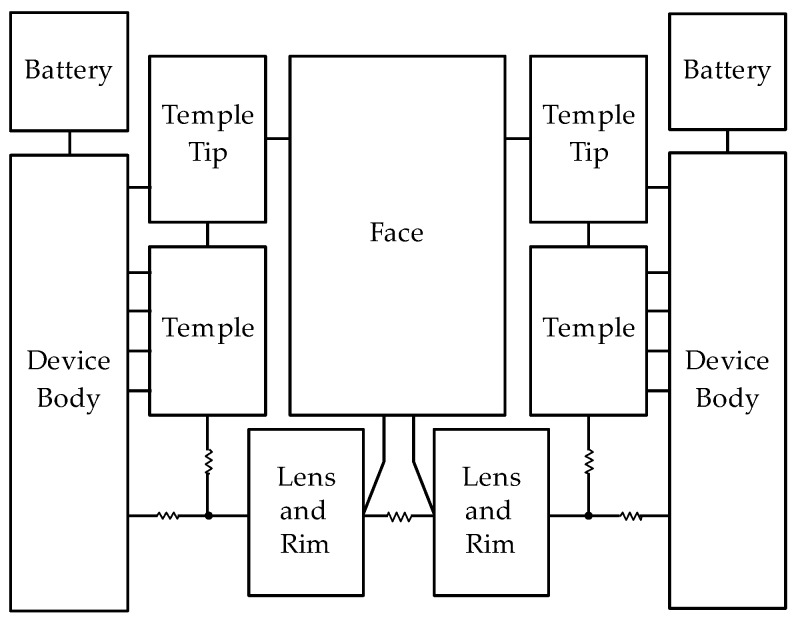
Block diagram for entire thermal network of smart glasses.

A block diagram for the entire thermal network of smart glasses is shown in Figure 9. By representing each block by the equivalent thermal resistance circuit, our model can be applied to various types of smart glasses. Designers can remove or replace blocks when necessary. In this paper, a thermal model of each block is constructed by a representative example. By changing the thermal model of each block, smart glasses under various conditions can be expressed.

### 3.2. Basic Thermal Resistance Model

**Figure 10:**
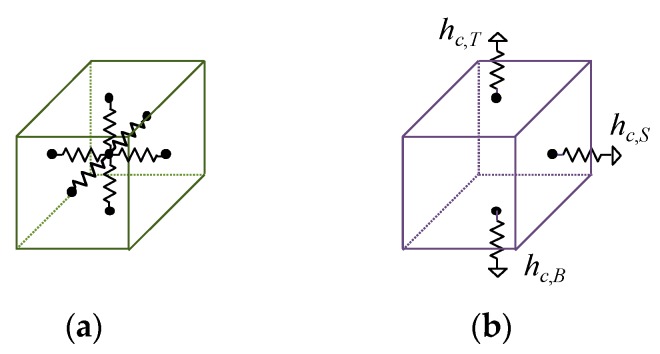
Thermal resistance circuits: (**a**) 3D heat conduction resistance model for one thermal cell, and (**b**) heat convection resistance model with convection heat transfer coefficients.

For a thermal resistance model for heat conduction of one cell (called a thermal cell), we basically use the three-dimensional (3D) equivalent resistance model shown in Figure 10a. The heat conduction resistance value in each segment can be calculated from the equation below.

(4) $$R=\frac{l}{kS}$$

where *l* is the length of a heat transfer path, *k* is the thermal conductivity of the material, and *S* is the cross-sectional area. The natural convection heat transfer coefficient value for air can be calculated from the equation below.

(5) $$h_{c}={\left(\frac{k^{4}\times g\times \beta \times Pr}{\eta^{2}}\right)}^{0.25}\times K\times {\left(\frac{\Delta T}{L}\right)}^{0.25}$$

where *g* is the acceleration of gravity, *β* is the air thermal expansion coefficient, *Pr* is the Prandtl number, *η* is the air kinematic viscosity, *K* is the coefficient in the vertical or horizontal direction, *ΔT* is the temperature difference, and *L* is the characteristic length [61]. The heat convection resistance value can be calculated from the equation below.

(6) $$R_{c}=\frac{1}{h_{c}S}$$

where *S* is the heat dissipation area. The convection heat transfer coefficients for the top, bottom, and side are distinguished, as shown in Figure 10b. The thermal resistance model for each part is created by setting thermal cells in heat transfer paths. The number of thermal cells depends on heat flow rates on the heat transfer path. The heat convection resistances are connected to the thermal cells in contact with ambient air. The ambient temperature is connected to a circuit ground.

### 3.3. Thermal Model of the Temple

**Figure 11:**
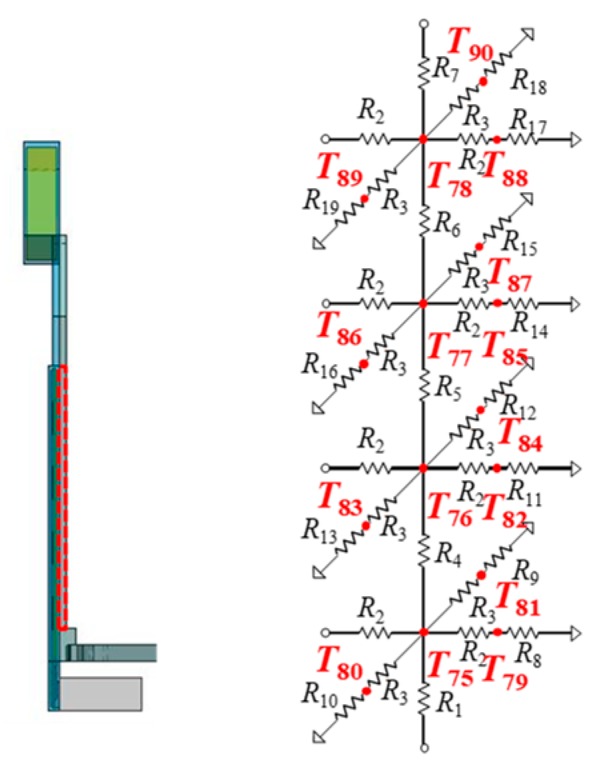
Thermal model of temple (at right side).

**Figure 12:**
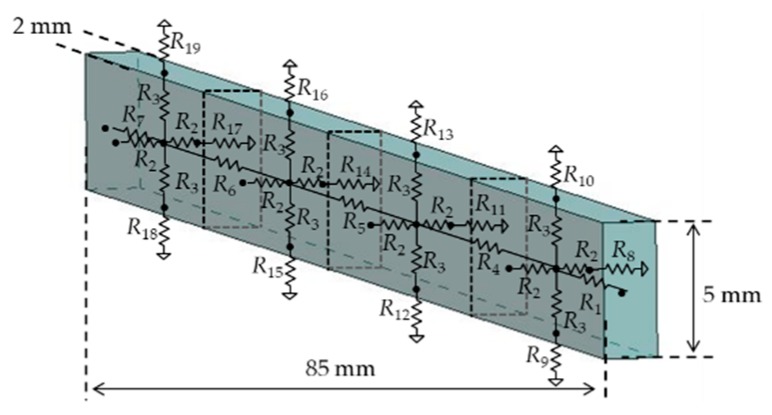
Thermal resistance circuits with a physical structure of the temple.

A temple of the glasses is divided into four cells to enable the connections with four heat generating components of a device body (see Figure 11). In this case, the method to derive resistance values of the thermal model is described in detail. Figure 12 shows thermal resistance circuits with the structure of a glasses’ temple. The temple is divided into four thermal cells. A thermal cell consists of the 3D heat conduction resistance model in Figure 10a, which has six resistances in three dimensions from the center of the thermal cell. For simplification, series resistance is expressed by one resistance. Heat conduction resistance values are calculated by substituting the length, thermal conductivity of the material, and area into Equation (4). For example, *R*_3_ in the vertical direction of one cell is 0.25 K/W from *l* = 2.5 mm, *k* = 236 W/mK, and *S* = 85/4 × 2 mm^2^. Table 4 summarizes the heat conduction resistance values for a temple.

The heat dissipations are set to three points at the back, top, and bottom of each cell of a temple. Heat convection resistance values are calculated by using Equations (5) and (6). The thermal profile for air shown in Table 5 is used for Equation (5). The characteristic lengths for heat dissipation areas use the short side for the horizontal surface and height for the vertical surface. The temperature difference is obtained by iterating the temperature calculation. The number of iterations used in this paper is three. As a concrete example to calculate the resistance in the vertical direction in one cell of a temple, the characteristic length is 2 mm, the heat dissipation area is 21.25 mm × 2 mm, and *K* is 0.52. When the temperature difference is 10 °C, the heat convection resistance becomes *R*_c_ = 2.14 × 103 K/W. Table 6 summarizes the heat convection resistance values of a temple. The top and bottom of a temple are connected with the bottom of a temple tip and with the rim, respectively.

### 3.4. Thermal Model of Electronic Device Body

**Figure 13:**
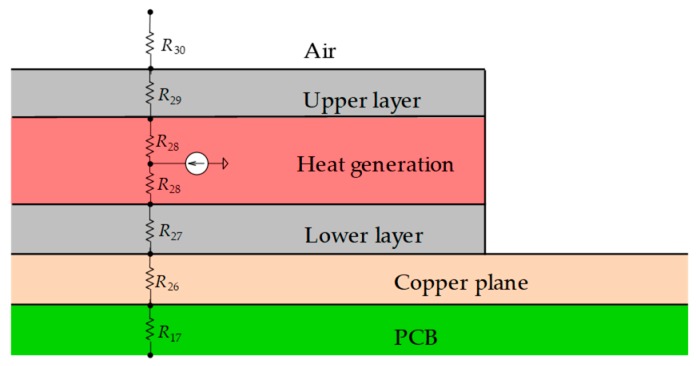
Thermal model around the heating component.

**Figure 14:**
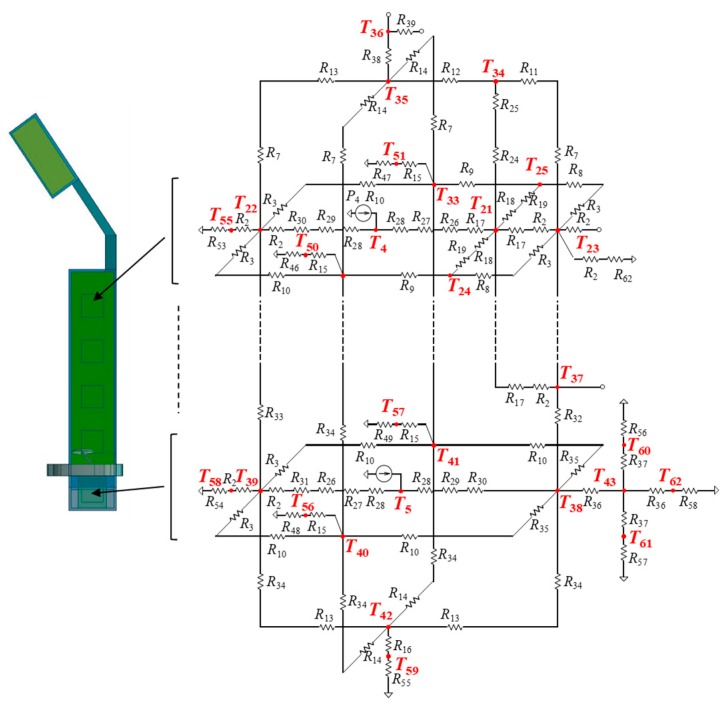
Thermal model of the device body (on the right side).

Figure 13 shows a thermal model around a heating component, which is composed of thermal resistances for each layer in the vertical direction and a heat source connected in the center of a heat generation layer. The heat source is given by power dissipated by a heat generating component. A power consumption value (in Watt) is given to the heat source. A thermal model of an electronic device body that incorporates five heat generating components is shown in Figure 11. It is a model for the device body of the right side. The device body is composed of heat generating components, PCBs, copper planes, device body cases, and a projector. Their parts are replaced by thermal cells. For example, a PCB is replaced with four thermal cells. The thermal resistance values in each thermal cell are calculated from the length, thermal conductivity, and cross-sectional area of a cell, as shown in Equation (4). The heat dissipations are set to four points at the front, back, top, and bottom of each heat generating component. A heat generating component represented in Figure 6 is modelled to the equivalent thermal resistance circuit in Figure 14. The heat source is located in the center with a current source symbol. Five heat generating components are arranged in the body case.

### 3.5. Thermal Model of Temple Tip

**Figure 15:**
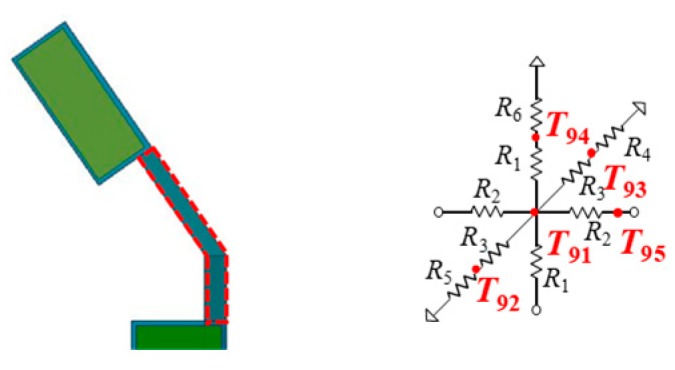
Thermal model of temple tip (on the right side).

A temple tip of the glasses is modelled as one thermal cell. Figure 15 shows a thermal model for a temple tip of the right side. The temple tip is expressed by the 3D equivalent resistance model shown in Figure 10a. The heat dissipations to an air are set to three points at the top, bottom, and back of a temple tip.

### 3.6. Thermal Model of Battery

**Figure 16:**
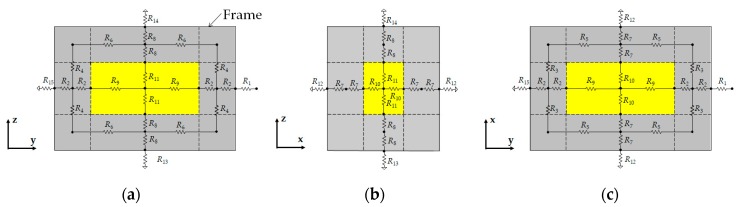
Thermal resistance circuits with a physical structure of the battery: (**a**) zy direction, (**b**) zx direction, and (**c**) xy direction.

**Figure 17:**
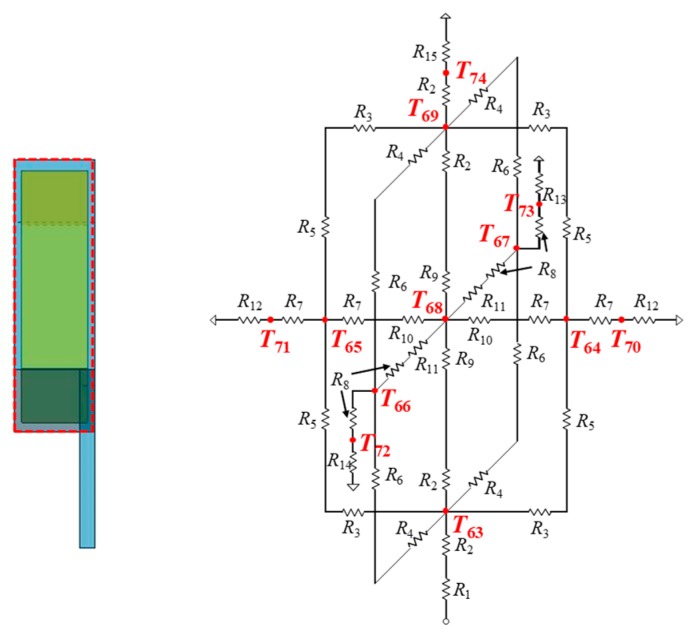
Thermal model of the battery (on the right side).

For a battery model, the inner liquid fluid and outer frame are represented by thermal resistances. Figure 16 shows thermal resistance circuits of a battery structure. R_1_ is the resistance to connect with a device body, R_2_ to R_8_ are the resistances of a frame, R_9_ to R_11_ are the resistances of the inner parts, and R_12_ to R_15_ are the heat convection resistances. Figure 17 shows the thermal model of the battery on the right side. The number of thermal cells used for a battery in this paper is seven. The heat dissipations are set to five points at the front, back, top, bottom, and tip of each cell of a battery.

### 3.7. Thermal Model of Lens and Rim

**Figure 18:**
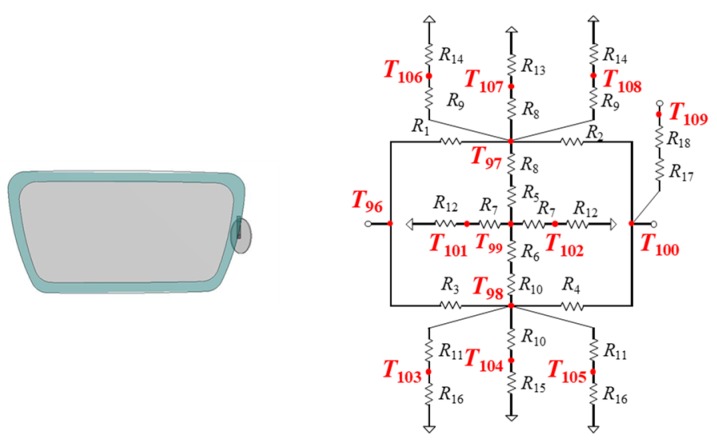
Thermal model of the lens and rim (on the right side).

A lens is represented by a two-dimensional thermal resistance model, and the rim of the lens frame is represented by a one-dimensional thermal resistance model. The lens and rim are divided into three parts: lens, upper rim, and lower rim. Three thermal cells are used. Figure 18 shows the model of the lens and rim of the right side. The heat dissipations are set to eight points at the front and back of a lens and the upper front, back, and top and the lower front, back, and bottom of a rim. The temperature of a nose pad on the nose is represented by T_109_. The node (T_109_) is connected with a face part.

### 3.8. Thermal Model of the Face Part

**Figure 19:**
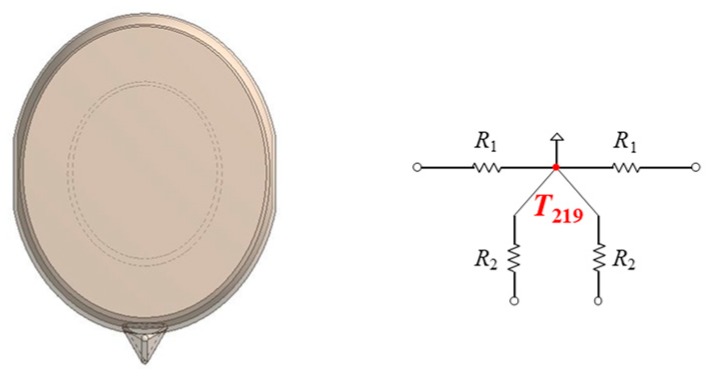
Thermal model of the face part.

Figure 19 shows a thermal model of a face for smart glasses. The face part is expressed by one thermal cell. The center node is set to 36.6 °C. R_1_ and R_2_ represent the thermal resistances of the face skin. The side and bottom of the face part are connected with the temple tip nose pads, respectively.

## 4. Experimental Results

**Figure 20:**
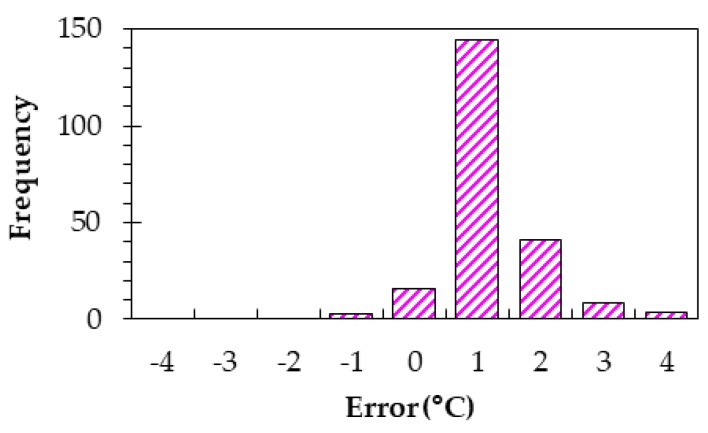
Error distribution for all nodes of the thermal network model.

We first verify the validity of our thermal model. Figure 20 shows a histogram in temperature differences between results obtained by our model and a thermal solver [62]. The absolute errors were almost within a few degrees. This is the result under the conditions that five heat generating components are placed at one side only and the power consumptions are uniform and 5 W in the total.

Next, we perform thermal analysis using the proposed model. Some countermeasures to reduce temperatures are shown in this section. It is important to reduce the surface temperature of smart glasses for more physical health safety and comfortable use.

**Figure 21:**
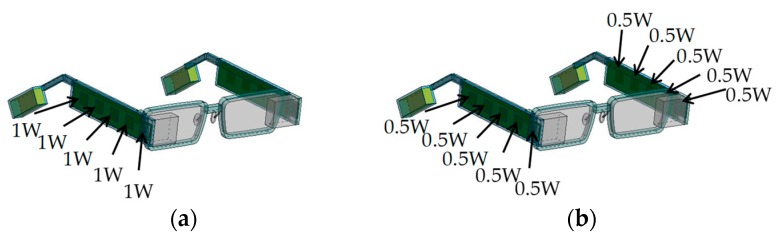
Illustration of heat sources: (**a**) one side and (**b**) both sides (example of total power of 5 W).

**Figure 22:**
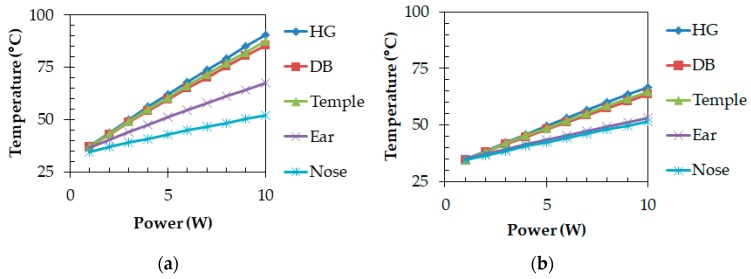
Temperature differences of heat sources: (**a**) one side and (**b**) both sides.

Figure 21 illustrates that heat sources (a) were placed on one side only and (b) were divided into both sides. Those are examples of conditions in which the total power consumption is 5 W and the power consumption is uniform. Figure 22 shows differences in temperatures when the device body was set to (a) one side only and to (b) both sides. In the figure, “HG” means the highest temperature in heat generating components, “DB” means the temperature on the surface of the device body, “Temple” means the temperature in the center of the temple frame, “Ear” means the temperature of the frame surface where the glasses frame is on the ear, and “Nose” means the temperature of a nose pad. In their parts, the temperature of the “Ear” and “Nose” are very important since they directly touch the skin. For example, under the condition of the total power consumption of 5 W, when the device body was set to one side only, the temperatures on the back side of the temple and the ear are 60.6 and 51.0 °C, respectively. On the other hand, when the device body was set to both sides, the temple and ear temperatures were 48.6 and 43.6 °C, respectively. By distributing the device body (heat generating components), the temperature rising to the ambient temperature of 25 °C can be reduced by 33.6% and 29.4%. The difference of the ear temperature from the core temperature of 36.6 °C is reduced by 51.4%. As seen from Figure 22, the temperature growths can be significantly suppressed by distributing heat sources regardless of total power consumption.

**Figure 23:**
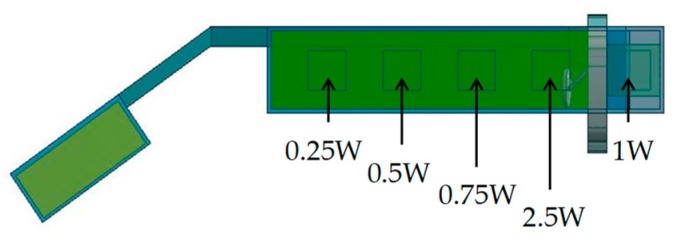
Device body with power consumptions in descending order at one side.

**Figure 24:**
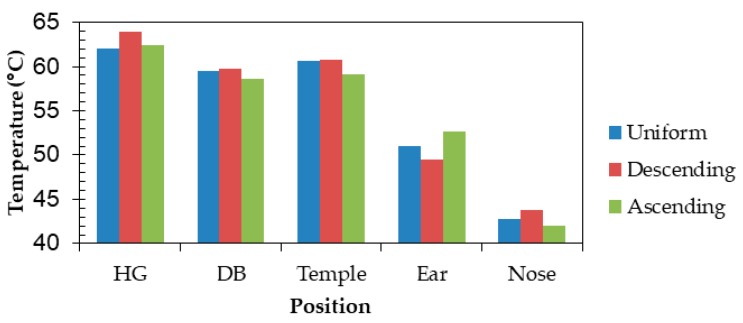
Effect of frame materials on temperature.

Figure 23 illustrates the conditions in which power consumptions are not uniform and heat sources were placed at one side only. Figure 24 shows temperature results at each part when power consumptions are uniform, in descending order, and in ascending order. The temperatures of “Ear” were 51.0, 49.4, and 52.7 °C (temperature differences from the core temperature of 36.6 °C were 14.4, 12.8, and 16.1 °C) for uniform, descending order, and ascending order. The descending order can reduce a temperature rise by 11.1% compared with uniform power consumptions. Therefore, the temperature of the part near the ear that directly touches the skin can be reduced when power consumptions of heat generating components are placed in descending order.

**Figure 25:**
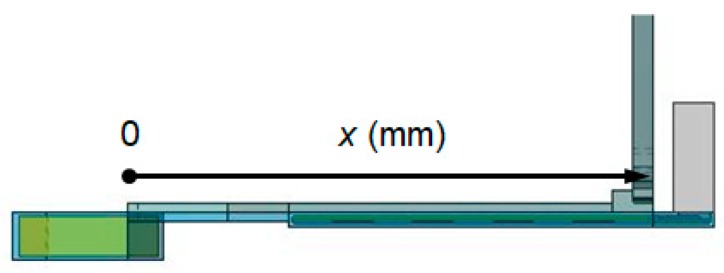
Illustration of the position on the temple.

**Figure 26:**
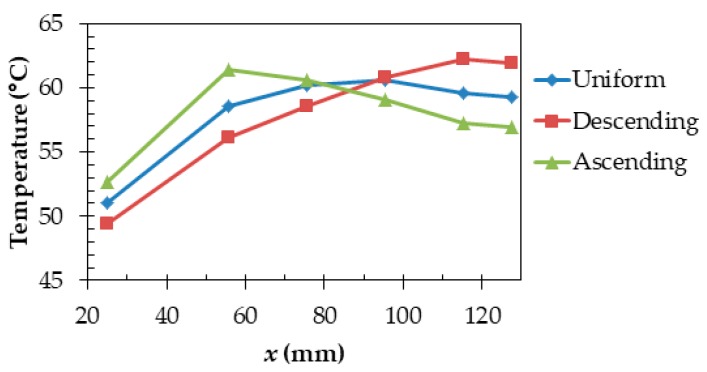
Difference in temperatures due to a position on the temple.

Figure 25 illustrates the position on the temple in the x direction. Figure 26 shows the temperatures at each position when power consumptions are uniform in descending order and in ascending order. For example, at 55.8 mm, temperatures for a uniform, descending order, and an ascending order are 58.6, 56.1, and 61.4 °C. The descending order is superior in that it can reduce the temperature near the ear.

**Figure 27:**
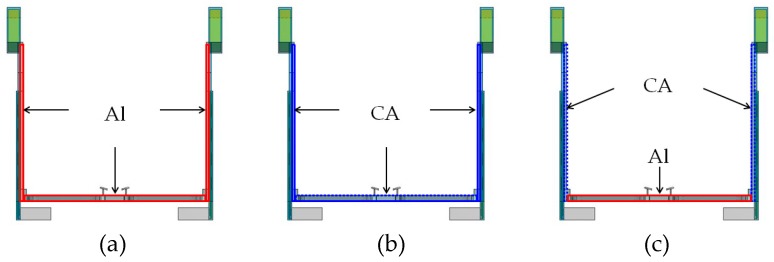
Illustration of frame materials: (**a**) Al, (**b**) CA, and (**c**) CA for the temple and temple tip and Al for others.

**Figure 28:**
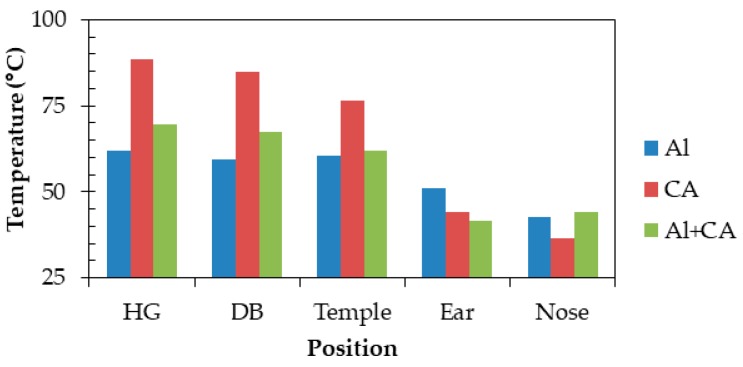
Effect of frame materials on temperature.

Figure 27 illustrates differences in the frame materials, where (a) is the all-Al frame, (b) is the all-CA frame, and (c) is the CA temples and temple tips and Al rims. Figure 28 depicts that Al greatly reduces temperatures of “HG,” “DB,” and “Temple,” whereas CA extremely raises the temperatures. The reason is thermal conductivity of Al is much higher than that of CA. However, the temperature of “Ear” is highest (51.0 °C) when Al was used but lowest (41.6 °C) when Al and CA were used. The temperature differences from the core temperature of 36.6 °C were 14.4 and 5.0 °C. The combination can suppress a temperature rise by 65.3% compared with Al only. For parts directly contacting the body, we found that a combination of CA temples and temple tips and Al rims is the best. The result obtained by a combination of Al and CA can satisfy the limit temperature (43 °C), which does not cause a low temperature burn [32]. Therefore, the method will be effective for the physical health safety and comfortable use of future smart glasses.

## 5. Discussions

### 5.1. Validity of Thermal Network Model

**Figure 29:**
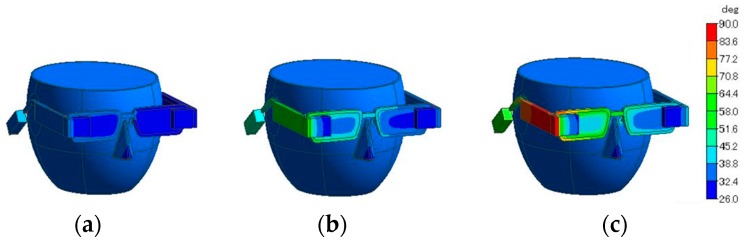
Temperature distributions when power consumptions are (**a**) 1 W, (**b**) 5 W, and (**c**) 10 W.

**Figure 30:**
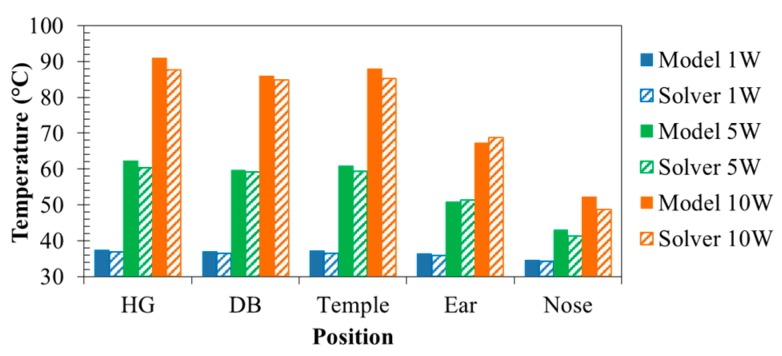
Comparison of results by the model with results given by the solver.

The accuracy of the proposed thermal network model was verified by a finite element method (FEM)-based 3D thermal solver. Figure 20 in Section 4 shows the result of errors at all nodes when power consumption was 5 W. Figure 29 shows temperature distributions obtained by a solver when power consumption varied. Figure 30 compares the model and a commercial solver in the temperature at the main nodes when power consumptions were 1, 5, and 10 W. We can see that differences in temperature between the model and solver hardly occur.

Simulation-based design and optimization of an accelerometer subject to thermal loads has been presented [63]. As verification of thermal simulations, comparisons of simulations and measurements in smartphones, a power semiconductor device, and a tablet device have been reported [60,64,65,66,67]. The simulation results agree with the measurement results. This means that if data to be inputted into a solver are near real data, the model corresponding to simulation results can reproduce temperature characteristics of real products. Thus, the verification of the proposed model by measurements is our future plan.

This paper presents a thermal network model in a steady-state condition for the entire thermal network of smart glasses. There are two types of thermal analysis: steady-state (or static) and transient (or dynamic). In this work, we are interested in the temperatures in the steady state rather than ones in transient time since smart glasses are designed to be worn for a long time. Steady-state thermal simulation results have been presented [60,64,65,66,67].

In general, advanced systems operate in the range of the ns to μs-order because of circuit frequencies of MHz to GHz-order. Power consumptions also change within the same range. On the other hand, time until the temperature of each node of smart glasses used in this work is stabilized in in the range of the ms to min-order. In steady-state thermal analysis, a constant power (e.g., average power) can be used [60,66,67].

### 5.2. Advantages of Thermal Network Model

The previous works related to this paper and the advantages of the proposed thermal network model are discussed in this sub-section. There have been several technical reports of thermal modeling for electrical and electric equipment [62,65,67]. These methods can be used for thermal simulations. The thermal resistance network is commonly composed of thermal cells with thermal conduction resistances, convection heat transfer coefficients, and heat sources. However, the thermal resistance network is specialized for smart glasses and is very simple because the number of elements is reduced. To the best of our knowledge, no technical report of thermal models and countermeasures for smart glasses exists. Moreover, thermal simulations of smart glasses have hardly been reported.

The thermal network model was implemented in Microsoft Visual Basic. The advantages of using the thermal network model are speed and convenience. The former speed is, for an example, that the runtime taken by using the thermal network model was about 11 sec, whereas that taken by using a thermal solver was about 337 sec. The model is, thus, more than 30 times faster. All experiments were run on an Intel Xeon CPU X5687 with 3.6 GHz. The latter (convenience) is that the model can easily estimate effects of structures and materials of parts on temperature. Moreover, by using the model, designers can optimize various parameters of future smart glasses.

## 6. Conclusions

In this paper, we have presented a thermal network model for thermal designs of future smart glasses. Thermal countermeasures for ensuring more safety and comfort have also been presented. Thermal analysis using the model demonstrates that plastic should be used for a part of the temples and temple tips for hanging on the ear. Al should be used for the other parts of the frame, and the device body should be located on the lens side and divided into both sides as much as possible.

## Figures and Tables

**Table 1:** Thermal properties of smart glasses.

Parts	Material	Abbreviation	Thermal Conductivity (W/mK)
Frame	Aluminum	Al	236
	Cellulose acetate	CA	0.2
Display	Polycarbonate	PC	0.19
Lens	Polycarbonate	PC	0.19
Nose pad	Cellulose propionate	CP	6
Battery	---	---	15

**Table 2:** Thermal properties and size of IC package.

Component	Thermal Conductivity (W/mK)	Thickness (mm)
Mold	0.88	---
Metal layer	98	0.003
Device layer	149	0.001
Si substrate	149	0.1
Bumps	60	0.08
Package substrate	149	0.2
Balls	33	0.35
PCB	13	0.8
Copper plane	401	0.03

**Table 3:** Thermal properties and size of liquid crystal on the silicon (LCOS) device.

Component	Thermal Conductivity (W/mK)	x, y, z (mm)
Glass substrate	0.8	8, 8, 0.5
Liquid crystal layer	0.15	8, 8, 0.005
Copper plane	401	14, 13, 0.03
PCB	13	14, 13, 0.8

**Table 4:** Heat conduction resistance values of the temple.

Variable	*l* (mm)	*S* (mm^2^)	Thermal Conductivity (W/mK)	Thermal Resistance (K/W)
*R*_1_, *R*_7_	10.625	10	236	4.50
*R*_2_	1	106.25	236	0.04
*R*_3_	2.5	42.5	236	0.25
*R*_4_, *R*_5_, *R*_6_	21.25	10	236	9.00

**Table 5:** Thermal profile for air.

Parameter of Air	Value
Thermal conductivity (W/mK)	2.625 × 10^−2^
Acceleration of gravity (m/s^2^)	9.80665
Thermal expansion coefficient (1/K)	3.247 × 10^−3^
Prandtl number	7.268 × 10^−1^
Kinematic viscosity (m^2^/s)	1.655 × 10^−5^
*K* in the vertical direction	0.56
*K* in the lower horizontal direction	0.26
*K* in the upper horizontal direction	0.52

**Table 6:** Heat convection resistance values of the temple.

Variable	*l* (mm)	*S* (mm^2^)	*K*	Thermal Resistance (K/W)
*R*_8_, *R*_11_, *R*_14_, *R*_17_	5	106	0.56	1.0 × 10^3^
*R*_9_, *R*_12_, *R*_15_, *R*_18_	2	42.5	0.26	4.29 × 10^3^
*R*_10_, *R*_13_, *R*_16_, *R*_19_	2	42.5	0.52	2.14 × 10^3^
